# The use of insecticide-treated nets for reducing malaria morbidity among children aged 6-59 months, in an area of high malaria transmission in central Côte d'Ivoire

**DOI:** 10.1186/1756-3305-3-91

**Published:** 2010-09-22

**Authors:** Benjamin G Koudou, Hala Ghattas, Clémence Essé, Christian Nsanzabana, Fabian Rohner, Jürg Utzinger, Brian E Faragher, Andres B Tschannen

**Affiliations:** 1 Vector Group, Liverpool School of Tropical Medicine, Pembroke Place, Liverpool L3 5QA, UK; 2 Centre Suisse de Recherches Scientifiques en Côte d'Ivoire, 01 BP 1303, Abidjan 01, Côte d'Ivoire; 3 UFR Sciences de Nature, Université d'Abobo-Adjamé, 02 BP 801, Abidjan 02, Côte d'Ivoire; 4 MRC International Nutrition Group, London School of Hygiene and Tropical Medicine, Keppel Street, London WC1E 7HT, UK; 5 Department of Nutrition and Food Sciences, American University of Beirut, P.O. Box 11-0236, Beirut 1107 2020, Lebanon; 6 Institut d'Ethno Sociologie, Université de Cocody-Abidjan, 01 BP V34, Abidjan 01, Côte d'Ivoire; 7 Department of Epidemiology and Public Health, Swiss Tropical and Public Health Institute, P.O. Box, CH-4002 Basel, Switzerland; 8 University of Basel, P.O. Box, CH-4003 Basel, Switzerland; 9 Institute of Food, Nutrition, and Health, Swiss Federal Institute of Technology Zurich, CH-8092 Zurich, Switzerland; 10 Clinical Group, Liverpool School of Tropical Medicine, Pembroke Place, Liverpool L3 5QA, UK

## Abstract

**Background:**

Long-lasting insecticidal nets (LLINs) are an important tool for controlling malaria. Much attention has been devoted to determine both the effect of LLINs on the reduction of *Plasmodium *infection rate and on clinically-confirmed malaria cases in sub-Saharan Africa. We carried out an epidemiological study to investigate whether LLINs impact on *Plasmodium *prevalence rate and the proportion of clinically-confirmed malaria cases, in five villages in the district of Toumodi, central Côte d'Ivoire.

**Methods:**

From April 2007 to November 2008, a community-based malaria control programme was implemented in the study villages, which involved large-scale distribution of LLINs, and training and sensitization activities within the community. We determined the effect of this programme on *Plasmodium *prevalence rate, clinically-confirmed malaria cases and proportion of high parasitaemia rates in children aged 6-59 months through a series of cross-sectional surveys starting in April 2007 and repeated once every 6 months.

**Results:**

We observed a significant decrease in the mean *P. falciparum *prevalence rate from April 2007 to April 2008 (p = 0.029). An opposite trend was observed from November 2007 to November 2008 when *P. falciparum *prevalence rate increased significantly (p = 0.003). Highly significant decreases in the proportions of clinical malaria cases were observed between April 2007 and April 2008 (p < 0.001), and between November 2007 and November 2008 (p = 0.001).

**Conclusions:**

Large-scale distribution of LLINs, accompanied by training and sensitization activities, significantly reduced *Plasmodium *prevalence rates among young children in the first year of the project, whereas overall clinical malaria rates dropped over the entire 18-month project period. A decrease in community motivation to sleep under bed nets, perhaps along with changing patterns of malaria transmission, might explain the observed increase in the *Plasmodium *prevalence rate between November 2007 and November 2008.

## Background

In Côte d'Ivoire, malaria remains the primary cause of health seeking at dispensaries, particularly during and shortly after the rainy season (from approximately mid-August to mid-October), accounting for 42% of all visits [1]. Moreover, malaria is the leading cause of hospitalization (70%) and mortality (15%), as recorded at paediatric units of hospitals [1].

With regard to malaria prevention, insecticide-treated nets (ITNs) have a track record of reducing malaria-related morbidity and mortality [2], and hence are increasingly being utilized in sub-Saharan Africa and other malaria-endemic areas. For example, sleeping under an ITN can decrease severe malaria by 45%, reduce premature birth rates by 42%, and significantly lower all-cause child mortality [3]. Unfortunately, in Côte d'Ivoire, ITN coverage is low (i.e. only 6% of children aged below 5 years slept under an ITN according to the 'multiple indicators cluster survey' carried out in 2006) [4], with most of the nets in use being 'untreated'. However, to remain effective, nets need to be re-treated after several months, but re-treatment requires additional skills, and technical knowledge together with affordable products and community participation [5]. Inadequacy of these and other factors may hamper the expansion of large-scale programmes involving insecticidal nets, and hence re-impregnation rates remain low and the nets become ineffective [6]. Consequently, several types of long-lasting insecticidal net (LLIN) treatments have been developed and evaluated in a wide range of situations [7].

Research carried out in rural Tanzania showed that a social marketing campaign with key messages and products readily adapted to the local settings can boost the coverage rates of ITNs, result in significant health gains and represents a cost-effective intervention to prevent malaria-related death [8-10]. In view of these findings and given the low coverage rates of ITNs in Côte d'Ivoire, we were motivated to implement a community-based malaria control programme comprising of selective and mass distribution of LLINs in a highly endemic area of central Côte d'Ivoire [11]. In an initial evaluation, we assessed the exact number of households and age distributions of the population living in the study area. Common knowledge and perceptions pertaining to malaria prevention and use of LLINs were determined by using socio-anthropological surveys to collect baseline information prior to net distribution. Community health workers (CHWs) were trained to help mothers or caregivers to manage uncomplicated malaria cases and to sensitize community members on the advantages of sleeping under a LLIN. Of note, CHWs were carefully selected among the local community. They were proposed by village committees and issues of availability, commitment, honesty and willingness to participate were taken into account. CHWs were trained by community nurses in providing primary health care.

The goal of the project reported here was to reduce malaria morbidity in young children by promoting the use of LLIN within households, facilitated by CHWs. The study was implemented in five villages located in central Côte d'Ivoire that are characterized by the same climate and agricultural activities. We report a descriptive study with no defined control groups, using a 'before-after' evaluation design. Particular emphasis was placed on *Plasmodium *prevalence rate and the number of clinical malaria cases, determined by repeated cross-sectional surveys. *Plasmodium *transmission by malaria vectors was investigated through entomological surveys in parallel to parasitological surveys with details presented elsewhere [12].

## Methods

### Study area

**Figure 1:**
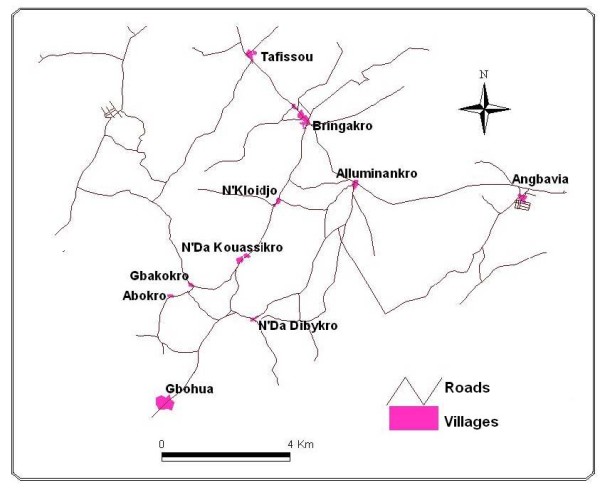
**Location of the five study villages in the health district of Toumodi, central Côte d'Ivoire**.

The study was conducted in five villages in the health district of Toumodi, in the commune of Djekanou in central Côte d'Ivoire: Tafissou-Bringakro (Taf-Bri), Gbohua-Angbavia (Gbo-Ang), Alluminankro-N'Kloidjo (All-N'Kl), N'Da Dibykro-N'Da Kouassikro (N'Da-N'Da) and Gbakokro-Abokro (Gba-Abo) (Figure 1). According to the house-to-house census carried out by interviewing heads of households in April 2007, the population of the target area was estimated at 7420 inhabitants.

The mean annual precipitation in this area is slightly above 1150 mm, and the mean annual temperature is 26.5°C. There are two seasons; a rainy season between April and October, and a dry season between November and March. The main activity in these communities is subsistence agriculture, supplemented with some cash crop, mainly consisting of cocoa, coffee and rubber production. The nearest health infrastructure is located in Taf-Bri, which is run by a nurse and three assistant nurses. Four of the villages have a primary school each (Taf-Bri, Gbo-Ang, N'Da-N'Da and Gba-Abo). At the time of our study, access to the power grid was available in two villages only (Taf-Bri and Gbo-Ang). Four villages had access to running water (Taf-Bri, Gbo-Ang, All-N'Kl and N'Da-N'Da).

Two entomological surveys were carried out in April and July 2008, after net distribution to householders. We recorded biting rates and entomological inoculation rates (EIRs) for *Anopheles gambiae s.s*., the main malaria vector in central Côte d'Ivoire [13-16]. The mean biting rates recorded were 1.5 and 4.1 bites/person/night, in April and July 2008, respectively. The corresponding mean EIR recorded were 31.4 and 147.8 infective bites/person/year in 2008. No mosquitoes from the species of *An. funestus *and *An. pharoensis *were found to be infected (BG Koudou, unpublished data).

The mean knock down (KD) and mortality rates recorded after performing standard World Health Organization (WHO) Pesticide Evaluation Scheme (WHOPES) tube bioassay tests in October 2008 with DDT, deltamethrin and permethrin on *An. gambiae *collected in this study area showed very high efficacy of these compounds. For example, we recorded a high KD rate with low fluctuations (range between 96.7% and 100%) and also a high mortality rate (range between 91.6% and 100%). Hence, there is no indication of resistance among adult *An. gambiae s.s *mosquitoes in the study area.

### Ethical approval and informed consent

Our study was approved by the Ivorian Ministry of Health through the national malaria control programme (NMCP). The heads of households in the study villages were informed about the objectives and procedures of the study and the parents or legal guardians of participating children gave written informed consent. Patients with malaria-related symptoms were treated free of charge according to the national malaria policy (i.e. artesunate plus amodiaquine) regardless of whether or not they participated in the cross-sectional surveys.

### Baseline evaluation

In April 2007, in parallel with a cross-sectional clinical survey, a questionnaire was administered to the heads of households in the five villages. We employed a pre-tested questionnaire to obtain data on demographics (number of individuals living in a household, age, sex and religion), professional and economic activities of the adult population, assessment of construction material for walls and roofing of houses, connection to the power grid, and appraisal of wealth indicators based on the presence of various assets (e.g. bicycle, radio, etc.). Householders were interviewed about major health problems, preventive measures against mosquito bites (e.g. use of ITNs, fumigating coils, etc.), the availability of latrines, and disposal of wastewater and solid waste. Additionally, in May 2007, a total of 16 focus group discussions (FGDs) were conducted with young men and women, adults and older individuals in three of the study villages (Taf-Bri, Gbo-Ang and All-N'Kl). Participants in the FGDs were recruited randomly with the help of CHWs. These FGDs centred on malaria causes and symptoms, and preventive measures employed against mosquito bites and commonly used antimalarial treatments.

Malariometric measurements were carried out in April 2007 before net distribution in the study villages to assess malaria parasitaemia and clinical cases in children aged 6-59 months. A finger prick blood sample was taken, thick and thin blood films were prepared on microscope slides and they were air-dried on the spot. The slides were transferred to a nearby laboratory and processed as detailed below.

### LLINs distribution and re-treatment of nets used during more than 30 months

In July 2007, we received 3600 LLINs (PermaNet 2.0) from Vestergaard-Frandsen, of which approximately 2800 nets were distributed in the study villages. The remaining 800 nets were distributed in small hamlets ("campements") that surround the study villages. LLIN distribution started in early August 2007 and was continued until October 2008. Heads of households were encouraged to use the nets for children younger than 5 years of age in order to protect them from mosquito bites. In July 2007, in collaboration with the non-governmental organization Stop Malaria International (SMI), all nets previously in the area and used for more than 30 months were treated free of charge with a long-lasting insecticide treatment (ICON MAXX; Syngenta Corporation). Heads of households were invited to send nets to a designated community location and the CHWs were trained by SMI to treat the untreated nets. Overall, 90 nets were treated according to the manufacturer's procedures.

### Sensitization and training activities

#### Sensitization activities

Subsequent to the initial evaluation, a series of sensitization campaigns (individual and mass sensitization) were conducted in each village. The aim of these campaigns was to improve knowledge on malaria transmission and preventive measures, and to increase acceptance of LLINs by the beneficiary communities. The activities started with a mass sensitization campaign that focused on the promotion of LLINs by the CHWs. In each village, CHWs held two meetings with the communities, during which everyone was invited to a designated location where the advantages of sleeping under a LLIN were explained and each householder received additional information to ensure that LLINs are used properly. Regular follow-ups were held in the intervention zones after net distribution. Communities were also informed about the importance of re-treating old nets. The sensitization campaign started in July 2007 and ended after reaching a high coverage level in March 2008 (i.e. more than half of children below the age of 5 years slept under a LLIN or a re-treated old net).

LLINs were ordered and packaged according to community preferences determined during an initial social survey carried out in April 2007. In agreement with village authorities, the CHWs were identified as net sellers. LLINs were sold at a low price (approximately US$ 2, i.e. a third of the costs for a net in Côte d'Ivoire at the time of our study).

#### Training activities

Two training activities were carried out in June and August 2007. First, with the support of the NMCP and the regional health district, CHWs were trained on the causes of malaria transmission, preventive measures against mosquito bites and the management of uncomplicated malaria cases among children under the age of 5 years. Training focussed on the recognition of typical symptoms of uncomplicated malaria, as well as severe symptoms warranting referral to a nearby health centre. Secondly, the CHWs were also trained to perform a follow-up of severe malaria cases, to act as intermediaries between the health services and the community, and to provide advice to mothers/caregivers of under-5 year-old children on how to manage uncomplicated malaria cases. The activities related to mothers' and caregivers' practices and behaviours in response to malaria attacks were assessed by an MSc student specializing in socio-anthropology who was in charge of this appraisal.

Antimalarial drugs (i.e. artemisinin-based combination therapy (ACT)) were kindly provided by a pharmaceutical company (Dafra Pharma; Turnhout, Belgium) and administered free of charge. Drugs were packaged in plastic bags labelled for one age group (0-59 months) according to standard recommendations by WHO. During the survey, children with measured fever (axillary temperature > 37.7°C) and infected with *P. falciparum *were treated free of charge, and ACTs were given to CHWs to treat new uncomplicated malaria cases detected in agreement with the local nurse after the departure of our research team. CHWs kept drugs and supplied them to mothers/caregivers on demand for treating new uncomplicated malaria cases. CHWs supervised and monitored mothers' and caregivers' practices and behaviours directly by following them while they treated malaria cases that occurred in the household. A part of the money collected when nets were sold was used to assure that ACTs were available during the length of the study.

### Cross-sectional surveys

Four repeated cross-sectional surveys were carried out in the study villages to assess malaria parasitaemia and clinical malaria in children aged 6-59 months. The surveys were done always in April and November, both in 2007 and 2008, i.e. once before the distribution of LLINs in August 2007 and three times afterwards. During the surveys, mothers and caregivers of under 5-year-old children were invited to accompany their children to a designated community location where a finger prick blood sample was taken from each child. Previously, all mothers who had one or several children below the age of 5 years were listed by the CHWs. During each cross-sectional parasitological survey, previously selected children aged 6-59 months came to a designated place with their mothers or caregivers and the children were examined, adhering to a standard protocol.

In the laboratory, air-dried thick and thin blood smears were stained with Giemsa for 45 min. Slides were examined by the same experienced laboratory technician throughout the study under a microscope at high magnification (x 100). *Plasmodium *species and gametocytes were identified and counted against 200 leucocytes. In case fewer than 10 parasites were found, the reading was continued for a total of 500 leucocytes. Parasitaemia was expressed by the number of parasites per μl of blood, assuming a standard count of 8000 leucocytes/μl blood. For quality control, 10% of the slides were randomly selected and re-examined by a senior laboratory technician.

In our study, all children having an axillary temperature > 37.7°C were considered as fever cases. Clinical malaria was defined as fever plus parasitaemia [17]. In all cross-sectional surveys, a measurement of axillary temperature was taken from every child aged 6-59 months. Children with clinical malaria were treated with the first-line ACT antimalarial (i.e. artesunate plus amodiaquine) and paracetamol. High parasitaemia was defined as > 5000 parasites/μl blood [15]. In each village, complicated malaria cases were referred to the nearest health centre with the help of the CHWs. During each cross-sectional survey, the mothers and caregivers of the children were interviewed about whether their children had slept under a LLIN the night before the survey. Before each survey, mothers and caregivers were invited to fill in and sign an informed consent form. The variation observed in the number of children examined is explained by the fact that during each survey, we examined children who were available and agreed to participate to the study.

### Follow-up of LLIN distribution campaign

Every 2 months, the coordinator of the project had meetings with the CHWs in order (i) to assess the number of new LLINs sold and (ii) to evaluate the number of new LLINs installed in household sleeping rooms. The goal of these meetings was to assess the new bi-monthly coverage rate of LLINs in each village, among children aged 6-59 months, and at the household level in general.

The monthly number of new LLINs sold was used to assess the overall number of LLINs sold. Additionally, every two months, CHWs assessed the number of LLINs installed and properly used in households. During the net promotion campaign, the number of LLINs sold per household was known because CHWs sold the LLINs only to heads of households. As the number of persons living in each household was determined beforehand, CHWs sold exactly the number of nets that were needed per household. Net coverage rates recorded in CHWs log-books were checked during two monitoring and evaluation visits in the study sites (April and October 2008).

### Statistical analysis

Data were analysed in STATA version 9.0 (STATA Corporation; College Station, TX, USA). Monthly malaria prevalence rates were computed for April 2007, November 2007, April 2008 and November 2008 for each village separately and then for all villages combined. These rates are reported with their 95% confidence intervals (CIs). As it was only possible to evaluate random samples of children in each village at each of the four assessment times, the CIs are finite population corrected (i.e. adjusted for the known population of each village). Overall monthly prevalence rates were compared before and after the distribution of the LLINs, using logistic regression models that were adjusted for the clustering of individuals within villages (by including village number as a dummy random variable, using the "cluster (*village code*)" subcommand in the "logistic" subroutine in Stata 9.0). To allow for known seasonal variation in malaria rates in the study area [16], comparisons were restricted to April 2007 *vs*. April 2008 and November 2007 *vs*. November 2008. No formal comparisons were made within individual villages as this was outside the remit of the study; differences between villages were adjusted for by treating these as clusters. Statistical significance was set at the conventional alpha = 0.05 (5%) level.

## Results

### Socioeconomic analyses and bed net use at baseline

Table 1 presents the results of the initial survey carried out in all households (n = 717) in each of the five villages. Improved sanitation facilities (modern latrines and electricity) are not widely available in the study area.

**Table 1:** Household characteristics and protective measures against mosquito bites in five study villages, central Côte d'Ivoire.

Socioeconomic indicators	Study village					Overall
	**Gbohua-Angbavia**	**Tafissou-Bringakro**	**N'Da Dibykro-N'Da Kouassikro**	**Alluminankro-N'Kloidjo**	**Gbakokro-Abokro**	

Number of households	245	158	127	120	67	717

Number of children ≤ 5 years	256	320	175	159	81	991

Houses without cement bricks (%)	56.2	50.7	31.3	55.7	0	43.1

Roof with thatched-grass (%)	16.8	23.6	11.6	23.5	6.1	17.4

Illiteracy rate (%)	64.1	53.3	53.8	62.1	46.2	58.1

Protective measures against mosquito bites						

Nets coverage rate (%)	2.5	3.6	2.4	7.2	1.6	3.2

Fumigating coils (%)	55.9	17.0	36.7	14.6	3.8	28.0

Use of insecticide sprays (%)	13.2	14.2	8.7	22.5	19.2	15.2

No protective measure	21.9	51.7	43.9	46.5	60.8	43.2

Preferred colour of bed net						

Blue (%)	46.9	44.4	48.4	38.9	57.7	45.3

Green (%)	24.7	22.1	22.6	37.8	15.4	22.8

White (%)	21.1	7.9	10.1	3.8	3.8	12.1

Other colours	3.2	13.7	8.8	9.6	13.2	9.6

Don't know	4.1	11.9	10.1	9.9	9.9	10.2

Contribution to pay for a LLIN (in F CFA)						

< 1000 (%)	1.8	4.2	23.4	1.6	6.5	7.5

1000 (%)	97.2	82.7	62.1	50.4	88.5	77.1

1500 (%)	1.0	11.1	9.1	37.8	3.8	12.4

>1500 (%)	0.0	2.0	5.4	10.2	1.2	3.8

Preferred shape of LLINs						

Rectangular (%)	55.1	76.4	55.6	67.3	88.5	64.1

Cylindrical (%)	41.8	13.1	18.2	11.4	7.7	23.4

Conical (%)	3.1	10.5	26.2	21.3	3.8	12.5

Convinced of LLINs efficacy (%)	93.5	92.2	72.2	57.9	46.2	84.7

The surveys showed a clear difference between reported ownership and net use. Though a third of households (34.2%) possessed a net, only 3.2% of householder reported that they actually used these nets (corresponding to 237 persons). A common reason stated by adults for not using a net was that the nets were not felt appropriate. The proportion of children below the age of 5 years who slept under a net was low (14.2%). With regard to personal protective measures against mosquito bites, fumigating coils was the most widely used tool (28.0%). Approximately one out of seven households used insecticide sprays, mainly applied on walls and ceilings (15.2%).

The preferred colour for the use of LLINs was blue; almost half of the interviewed households mentioned this colour (45.3%), followed by green (22.8%) and white (12.1%). The preferred shape of LLINs mentioned by the households is rectangular (64.1%). More than three-quarter of the householders interviewed (77.1%) agreed to pay F CFA 1000 (approximately US$ 2), corresponding to one third of the LLIN price at the national public health pharmacy at the time of our study.

### Socio-anthropological qualitative analyses

According to the FGDs, the main causes of malaria were the sun, tiredness due to field work and household work, inadequate nutrition, cold and constipation. Concerning the use of preventive measures against mosquito bites, the majority of respondents mentioned anti-mosquito measures such as ITNs, fumigating coils, modern and traditional insecticides.

With regard to malaria symptoms, fever was clearly the most important one, followed by loss of appetite, tiredness, abdominal discomfort and diarrhoea. Additionally, yellow eyes and yellow urine were frequently mentioned. Modern health care providers interviewed moreover indicated constipation, diarrhoea, nausea, body aches and pains.

Traditional medicine is primarily sought when a malaria episode occurs. Modern drugs were used to a lesser extent, often simultaneously with traditional medicine.

### Impregnation of untreated nets, sensitization and training activities

A total of 11 CHWs in the five study villages were trained on malaria transmission, preventives measures against mosquito bites and the management of uncomplicated malaria cases among children aged 6-59 months.

With the support of SMI, CHWs were trained in the treatment of old nets. CHWs also served as intermediates between the health services and the community, and they were responsible for providing advice to mothers and caregivers of under-5 year-old children on how to manage uncomplicated malaria cases.

Following the sale of LLINs to household members, CHWs assisted in the installation of the LLINs. Fourteen months after net distribution, the proportions of children below the age of 5 years who slept under a LLIN were 47.3%, 56.4%, 51.1%, 55.4% and 50.2% in the villages of All-N'Kl, Gba-Abo, N'Da-N'Da, Taf-Bri and Gbo-Ang, respectively. Table 2 summarises the increase in net usage in young children during LLIN promotion campaigns in the study villages from April 2007 (baseline) to November 2008.

**Table 2:** Proportions of children under the age of 5 years who slept under a LLIN the night before a questionnaire survey in four cross-sectional surveys carried out between April 2007 and November 2008 (in bracket the number of children surveyed), in five villages in central Côte d'Ivoire.

Study sites	Proportions of children <5 years old sleeping under a LLIN			
	**April 2007**	**November 2007**	**April 2008**	**November 2008**

Gbohua-Angbavia	2.7 (169)	17.6 (174)	35.7 (81)	50.2 (125)
Gbakokro-Abokro	n.a	18.9 (108)	n.a	56.4 (69)
Tafissou-Bringakro	3.6 (262)	19.4 (192)	37.9 (166)	55.4 (146)
N'Da Dibykro-N'Da Kouassikro	2.4 (143)	16.2 (129)	34.5 (111)	51.1 (117)
Alluminankro-N'Kloidjo	7.2 (129)	17.3 (110)	33.6 (95)	47.3 (92)
Overall	3.5 (506)	17.9 (453)	36.0 (713)	52.1 (549)

### Malaria parasite infection rates

*P. falciparum *was clearly the predominant malaria parasite in the study villages. Its frequency among positive blood films obtained during the repeated cross-sectional surveys was above 87%. Single species infections with *P. malariae *were found in 3.7-6.2% of the positive slides. Mixed species infections with *P. falciparum *and *P. malariae *ranged between 1.2% and 3.8%. Neither *P. ovale *nor *P. vivax *single infections were diagnosed throughout the study. The annual gametocyte rates ranged between 2.4% and 3.7%.

#### *Plasmodium falciparum* prevalence rates

Table 3 summarises the results of the *P. falciparum *prevalence (number of positive children/number of children examined) obtained during repeated cross-sectional surveys carried out between April 2007 and November 2008, in the five study villages among children aged 6-59 months. The data revealed that the prevalence of *P. falciparum *was high in November 2007 and 2008 (during the short rainy season) and low in April 2007 and 2008 (beginning of the long rainy season).

**Table 3:** *Plasmodium falciparum *prevalence rates (95% confidence interval) and number of children examined per village (in bold), recorded during repeated cross-sectional surveys carried out between April 2007 and November 2008 in five study villages in central Côte d'Ivoire.

Study sites	Malaria parasitaemia prevalences (95% CI)^†^			
	**April**		**November**	
		
	**2007**	**2008**	**2007**	**2008**

Gbohua-Angbavia	45.6 (41.1-50.0)/**169**	54.3 (45.2-63.5)/**81**	62.6 (58.5-66.8)/**174**	74.4 (68.9-79.9)/**125**
Gbakokro-Abokro	n.a		70.0 (68.9-71.1)/**108**	88.1 (83.7-92.6)/**69**
Tafissou-Bringakro	47.7 (45.1-50.3)/**262**	25.3 (20.7-29.9)/**166**	62.0 (57.6-66.4)/**192**	82.9 (78.3-87.4)/**146**
N'Da Dibykro-N'Da Kouassikro	28.7 (25.5-31.9)/**143**	17.1 (12.8-21.4)/**111**	52.7 (48.2-57.2)/**129**	59.0 (53.8-64.2)/**117**
Alluminankro-N'Kloidjo	57.8 (54.5-61.1)/**129**	17.9 (12.9-22.9)/**95**	55.5 (50.2-60.7)/**110**	90.2 (86.2-94.2)/**92**
Overall	45.3 (43.6-47.0)/**506**	26.9 (24.2-29.7)/**453**	60.2 (58.3-62.3)/**713**	77.6 (75.3-79.8)/**549**
	April 2007 *vs*. April 2008: p = 0.029^‡^		November 2007 *vs*. November 2008: p = 0.003^‡^	

n.a: not assessed †: finite population corrected ‡: adjusted for cluster sampling design

An overall significant decrease in the *P. falciparum *prevalence occurred from April 2007 to April 2008 (p = 0.029), although it was noted that the rate increased in this period in one of the study villages (Gbo-Ang). An opposite trend was observed from November 2007 to November 2008; *P. falciparum *prevalence rate increased significantly (p = 0.003), with consistent increases observed in each of the five villages.

#### Proportion of high parasitaemia and use of LLINs

Table 4 summarises the proportion of high parasitaemia (> 5000 parasites/μl blood) between children who slept under a LLIN and those who did not in the night before the survey, stratified by study village.

**Table 4:** Proportion of children less than 5 years old with high parasitaemia levels (> 5000 parasites/μl blood; in brackets number of high parasitaemia out of the number of children examined), observed during repeated cross-sectional surveys carried out between April 2007 and November 2008 in five study villages in central Côte d'Ivoire.

Study sites	Periods of surveys carried out		P-value
	**Sleeping under a LLIN**	**Not sleeping under a LLIN**	

Gbohua-Angbavia	4.0 (11/275)	7.7 (21/274)	0.084
Gbakokro-Abokro	13.0 (13/100)	5.2 (4/77)	0.111
Tafissou-Bringakro	5.7 (24/424)	5.6 (19/342)	0.953
N'Da Dibykro-N'Da Kouassikro	2.9 (7/236)	6.1 (16/264)	0.115
Alluminankro-N'Kloidjo	4.6 (10/218)	6.7 (14/208)	0.365
Total	5.2 (65/1253)	6.3 (74/1165)	0.246

Sleeping under a LLIN did not have any effect on the proportion of high parasitaemia in the study villages. Indeed, the differences between the proportions of high parasitaemia recorded among children who slept under a LLIN and those who did not in the night before the survey showed no statistical significance: All-N'Kl (p = 0.246), Gba-Abo (p = 0.084), N'Da-N'Da (p = 0.365), Taf-Bri (p = 0.953) and Gbo-Ang (p = 0.111).

#### Proportions of fever cases found to be positive for *P. falciparum*

Table 5 summarises the proportion of fever cases found to be positive for *P. falciparum *after blood examination (clinical malaria cases). A highly significant fall in clinical malaria rates was observed both between April 2007 and April 2008 (p < 0.001), and between November 2007 and November 2008 (p = 0.001). These trends were reflected in all five villages.

**Table 5:** Percentage of children less than 5 years old examined positive after blood examination (number of positive fever cases/number of children examined), and number of children examined per village (in bold), recorded during repeated cross-sectional surveys carried out between April 2007 and November 2008 in five study villages in central Côte d'Ivoire.

Study sites	Proportions of clinical malaria cases (95% CI)^†^			
	**April**		**November**	
		
	**2007**	**2008**	**2007**	**2008**

Gbohua-Angbavia	17.2 (13.8-20.5)/169	3.6 (0.2-7.0)/81	12.5 (9.8-15.4)/174	5.6 (2.7-8.4)/125
Gbakokro-Abokro	n.a		6.3 (5.6-6.9)/108	3.3 (1.0-5.7)/69
Tafissou-Bringakro	18.3 (16.3-20.2)/262	6.3 (3.6-9.1)/166	10.4 (7.7-13.2)/192	8.3 (4.9-11.6)/146
N'Da Dibykro-N'Da Kouassikro	9.8 (7.7-11.9)/143	4.5 (2.1-6.8)/111	19.4 (15.8-22.9)/129	8.3 (5.1-11.6)/117
Alluminankro-N'Kloidjo	8.1 (6.3-10.0)/129	5.3 (0.2-8.2)/95	13.6 (10.0-17.3)/110	10.6 (6.6-14.7)/92
Overall	14.4 (13.2-15.6)/506	5.1 (3.7-6.5)/453	12.7 (11.3-14.0)/713	7.5 (6.0-9.0)/549
	April 2007 *vs*. April 2008: p < 0.001^‡^		November 2007 *vs*. November 2008: p = 0.001^‡^	

n.a: not assessed †: finite population corrected ‡: adjusted for cluster sampling design

## Discussion

The increase recorded in *Plasmodium *prevalence rate from November 2007 to November 2008 could be explained by the fact that during the latter period, conditions such as rainfall and humidity were more conducive to mosquito development. Generally, when environmental conditions are favourable (monthly rainfall up to 150 mm and humidity around 85%), more productive *An. gambiae *breeding sites are formed, contributing to the increase of *Plasmodium *infection [18]. Previous studies carried out in sub-Saharan Africa, in areas of stable malaria characterized by high EIRs, showed that the protective efficacy of LLINs was lower [2]. The reduction recorded in LLIN protective efficacy is confirmed by the fact that several studies performed in three malaria stable West African countries (Ghana, Côte d'Ivoire and Burkina Faso) reported very low reduction on *Plasmodium *prevalence rate (13% reduction when the control group did not have any nets and 10% reduction when the control group had untreated nets) [19-21]. Increased records in *Plasmodium *prevalence rate could also be due to the fact that LLINs were not properly used by householders, specifically because we recorded a reduction in malaria transmission in April and July 2008, 9 months after net distribution (unpublished data). During this low transmission and biting period, the perceived benefits of reduction in mosquito bites and of malaria were considered not to be important by household members. Recent studies carried out in Burkina Faso [22] showed that LLINs were not used during the dry season (period of low biting rates) and when the perceived benefits of reduction in mosquitoes bites and of malaria were considered not to be important, even during the high transmission period. Additionally, both studies have shown that the usefulness of LLINs in reducing malaria prevalence rate and morbidity was moderated by the fact that mosquitoes were considered to be only one of several factors which caused malaria [22].

From April 2007 to April 2008, a statistically significant reduction was observed in the overall *Plasmodium *infection rates recorded among children aged between 6 and 59 months. Indeed, activities carried out few months after net distribution by CHWs and mass sensitization campaigns undertaken in the study villages contributed to reduce drastically *Plasmodium *prevalence rate. It is conceivable that the motivation for the use of LLINs is high shortly after mass and household-to-household sensitization campaigns. This argument is confirmed by a recent study carried out in Burkina Faso showing that the motivation for the use of LLINs decreased after less than a year [22]. The increase in motivation for the use of LLINs could also be explained by the fact that nets were distributed during the short rainy season (from mid-September to mid-November), at a time *An. gambiae *biting rates are supposed to be high. During this study period, particularly in April, during the rainy season, previous studies carried out in three urban cities of West Africa (Abidjan, Cotonou and Ouagadougou) showed that sleeping under an ITN the night before the survey was protective against *Plasmodium *infection [23-25]. Additionally, a recent study showed that *Plasmodium *prevalence rate was strongly influenced by socioeconomic status, and the methods used for prevention depended on their perceived cost [26]. In our study, however, no link was found between the proportion of protective measures and *Plasmodium *infection rates, perhaps because in villages where we recorded the highest *Plasmodium *infection rates, the use of fumigating coils and insecticides was high.

Another important finding of our study is that we observed a significant decrease in the overall proportions of clinical malaria cases among children less than 5 years old during both study periods, from April 2007 to April 2008 and from November 2007 to November 2008. This observation might be explained by the considerable number of household members sleeping under LLINs instigated by the household-to-household and mass sensitization campaigns undertaken before and after net distribution. Of note, in this project, although the price of nets was relatively low, we did not carry out free distribution of nets. Similarly, in Tanzania, 10 years ago, social marketing of treated nets was identified as an effective means for malaria control in rural settings. Eighteen months after launching promotion of treated nets, 46% of 312 families with children aged less than 5 years reported that their children were sleeping under treated nets [8]. In a demographic surveillance system area of Tanzania, after a social marketing campaign, ITN coverage of infants rose from < 10% at baseline to > 50% some 3 years later. Treated nets were associated with a 27% increase in survival in children aged 1 month to 4 years [9]. A reduction in the number of clinical malaria cases was also recorded in Kafine, a village located in the northern rice-growing region of Côte d'Ivoire, where the main malaria vector, *An. gambiae s.s*., is resistant to permethrin and other pyrethroids [20]. In this village, the rate of malaria attacks was twice as high among non-users of mosquito nets. Meanwhile, previous studies performed in several West African malaria stable countries (Côte d'Ivoire, Gambia and Sierra Leone), the differences in treated nets protective efficacies against uncomplicated malaria were low (11%) [20,27,28].

Finally, in our study, no significant statistical difference was found between users and non-users of LLINs concerning the prevalence of high parasitaemia levels (> 5000 parasites/μl blood). This observation is in agreement with previous studies carried out in the savannah village of Kafine, northern Côte d'Ivoire, where no difference was found between user and non-user groups of mosquito nets with regard to the prevalence of high parasitaemia, or gametocytes or to the mean parasite load [20]. Generally, in a highly endemic area, there is no marked variation in malaria prevalence rates and morbidity during the season of high prevalence rate. Indeed, in some previous studies performed in East Africa treated nets did not appear to reduce malaria prevalence rate, parasite density and all cause morbidity [29,30]. Conversely, treated nets have proved to have a significant protective efficacy against high parasitaemia in Kenya among children less than 5 years [31].

To conclude, training and sensitization activities undertaken before and after net distribution contributed to reducing *Plasmodium *prevalence from April 2007 to April 2008 and to significantly reducing the number of clinical malaria cases. However, 8-9 months after net distribution, a decrease in the motivation for the use of nets probably favoured a significant increase in the overall *Plasmodium *prevalence rate from November 2007 to November 2008. Thus, continued long-term social marketing for promoting LLINs holds promise for effective malaria control in rural and urban malaria endemic countries.

## Competing interests

The authors declare that they have no competing interests.

## Authors' contributions

BGK: designed experiments, coordinated field activities, collected and analyzed data, wrote and revised the paper; HG: designed the study and participated in the coordination of the field activities; CE: designed experiments, collected data and analysed socio-anthropological data; CN: contributed to the study design and revised the paper; FR: designed the study, participated in the coordination of field activities and revised the paper; JU: participated in the design of the study and revised the paper; BEF: assisted with the statistical analysis and revised the paper; ABT: designed the study, participated in the coordination of field activities and revised the paper. All authors have read and agreed with the content of the submitted manuscript.
